# Glucose-6-Phosphate Dehydrogenase and NADPH Redox Regulates Cardiac Myocyte L-Type Calcium Channel Activity and Myocardial Contractile Function

**DOI:** 10.1371/journal.pone.0045365

**Published:** 2012-10-05

**Authors:** Dhwajbahadur K. Rawat, Peter Hecker, Makino Watanabe, Sukrutha Chettimada, Richard J. Levy, Takao Okada, John G. Edwards, Sachin A. Gupte

**Affiliations:** 1 Department of Biochemistry and Molecular Biology, University of South Alabama, Mobile, Alabama, United States of America; 2 Department of Medicine, Division of Cardiology, University of Maryland, Baltimore, Maryland, United States of America; 3 Departments of Physiology, Juntendo University School of Medicine, Tokyo, Japan; 4 Department of Physiology, New York Medical College, Valhalla, New York, United States of America; Instituto Nacional de Cardiologia, Mexico

## Abstract

We recently demonstrated that a 17-ketosteroid, epiandrosterone, attenuates L-type Ca^2+^ currents (I_Ca-L_) in cardiac myocytes and inhibits myocardial contractility. Because 17-ketosteroids are known to inhibit glucose-6-phosphate dehydrogenase (G6PD), the rate-limiting enzyme in the pentose phosphate pathway, and to reduce intracellular NADPH levels, we hypothesized that inhibition of G6PD could be a novel signaling mechanism which inhibit I_Ca-L_ and, therefore, cardiac contractile function. We tested this idea by examining myocardial function in isolated hearts and Ca^2+^ channel activity in isolated cardiac myocytes. Myocardial function was tested in Langendorff perfused hearts and I_Ca-L_ were recorded in the whole-cell patch configuration by applying double pulses from a holding potential of −80 mV and then normalized to the peak amplitudes of control currents. 6-Aminonicotinamide, a competitive inhibitor of G6PD, increased pCO_2_ and decreased pH. Additionally, 6-aminonicotinamide inhibited G6PD activity, reduced NADPH levels, attenuated peak I_Ca-L_ amplitudes, and decreased left ventricular developed pressure and ±dp/dt. Finally, dialyzing NADPH into cells from the patch pipette solution attenuated the suppression of I_Ca-L_ by 6-aminonicotinamide. Likewise, in G6PD-deficient mice, G6PD insufficiency in the heart decreased GSH-to-GSSG ratio, superoxide, cholesterol and acetyl CoA. In these mice, M-mode echocardiographic findings showed increased diastolic volume and end-diastolic diameter without changes in the fraction shortening. Taken together, these findings suggest that inhibiting G6PD activity and reducing NADPH levels alters metabolism and leads to inhibition of L-type Ca^2+^ channel activity. Notably, this pathway may be involved in modulating myocardial contractility under physiological and pathophysiological conditions during which the pentose phosphate pathway-derived NADPH redox is modulated (e.g., ischemia-reperfusion and heart failure).

## Introduction

Voltage-gated L-type Ca^2+^ channels play an important role in the regulation of myocardial contractile function by controlling Ca^2+^ entry and Ca^2+^-induced Ca^2+^ release from sarcoplasmic reticulum in cardiac myocytes. Their activity is modulated by a variety of neurotransmitters, hormones and autacoids via regulatory processes involving multiple enzymatic reactions. Among these modulators, the sex steroid 17β-estradiol attenuates L-type Ca^2+^ currents in isolated guinea-pig atrial [1] and ventricular [2] myocytes, while testosterone inhibits both native and human recombinant L-type Ca^2+^ channels from ventricular myocytes, single T-type Ca^2+^ channels from neonatal rat cardiomyocytes [3], [4], and both L- and T-type Ca^2+^ channels stably expressed in A7r5 and HEK 293 cells [5], [6]. The effects of both 17β-estradiol and testosterone are voltage-independent. By contrast, epiandrosterone, an inactive isomer of androsterone, attenuates L-type Ca^2+^ currents in isolated rat and rabbit ventricular myocytes in a voltage-dependent manner [7]. Although it is known that application of some steroids to cardiac myocytes shifts the current-voltage (I–V) relation and steady-state inactivation curve to more negative potentials, the mechanisms by which steroid hormones inhibit Ca^2+^ channel activity remain unclear.

The 17-ketosteroids [e.g., 17β-estradiol, testosterone, epiandrosterone and dihydroepiandrosterone (DHEA)] are known to inhibit glucose-6-phosphate dehydrogenase (G6PD), the rate-limiting enzyme in the pentose phosphate pathway (PPP), and to reduce intracellular NADPH levels [8]. We recently demonstrated that inhibition of G6PD by epiandrosterone or DHEA, an abundantly produced adrenal steroid, reduces NADPH levels in the isolated rat heart [7] and pulmonary and coronary arteries [9]–[11], exerts a negative inotropic effect in rat hearts [7], attenuates angiotensin II- and hypoxia-induced pulmonary vasoconstriction in isolated lungs [9], and relaxes isolated pulmonary and coronary arteries by partially opening K_v_ channels [9] and reducing levels of intracellular free Ca^2+^ [11]. Others have shown that DHEA inhibits G6PD, increases levels of oxidized glutathione, and diminishes Ca^2+^ transients in isolated rat cardiomyocytes [12]. Moreover, G6PD deficiency is common and there are point mutations found in this enzyme in different ethnic groups around the world, and individuals who harbor a Mediterranean-type mutation with mild deficiency are less likely to have cardiovascular diseases, including heart failure [13]. In contrast, individuals harboring a G6PD A mutation (African-type mutation) have high incidence of cardiovascular diseases [14]. Bearing these observations in mind, we hypothesized that a reduction in G6PD-derived NADPH may lead to inhibition of L-type Ca^2+^ channel activity, which is a key component of EC coupling, and decrease myocardial contractility. To test that idea and to shed light on the role played by G6PD and NADPH in regulating L-type Ca^2+^ channel and heart function, we studied the effects of 6-aminonicotinamide (6AN), a competitive G6PD inhibitor [15], and G6PD deficiency on cardiac metabolism and function, and L-type Ca^2+^ activity in isolated cardiac myocytes. We found that inhibition of G6PD caused small but significant reduction in metabolism, L-type Ca^2+^ currents, which are partially reversed by administration of exogenous NADPH, and cardiac function.

## Materials and Methods

This study was conducted in accordance with National Institutes of Health and American Physiological Society guidelines. The protocol was approved by New York Medical College (Protocol #98-12-0706), University of South Alabama (Protocol #11036) and University of Maryland (Protocol # 1009011) Animal Experimentation Committee. Experiments were performed with adult male Sprague-Dawley rats (288±24 g) purchased from Charles River Laboratories (MA, USA). Mice (17–18 wks old) were bred at New York Medical College, Valhalla, NY, USA and University of Maryland, MD, USA. The rats/mice were housed at the ambient room temperature and barometric pressure, were exposed to a 12∶12-h light-dark cycle, and were allowed free access to standard food and water. Male rats and hemizygous male and homozygous mice were used in this study. All chemicals and drugs used in this study were purchased from Sigma (MO, USA), unless otherwise noted.

### Measurement of Myocardial Function

Myocardial contractile function was determined in isolated hearts as reported in our previous paper [7]. Briefly, hearts were isolated and quickly perfused with Krebs buffer, pH 7.4, containing in mM [NaCl 116.0, NaHCO3 25.0, CaCl2 2.5, MgSO4 1.2, KCl 4.7, KH2PO4 1.2 and glucose 5.5] in constant flow mode. The hearts were stabilized for 45–60 minutes, and then perfused with 6-AN for 10 minutes after which 6AN was washed out for 60 minutes with normal Krebs solution. Left ventricular developed pressure (LVDP) was recorded by inserting a latex balloon in the left ventricle through mitral annulus and by inflating the balloon. End diastolic pressure was maintained at 10 mmHg. First derivatives +/− dP/dt were also determined. Data was collected and analyzed electronically using PowerLab software. Hemodynamic measurements were done continuously and data prior to perfusing the heart with 6AN solution (Pre) and 10 minutes after 6AN perfusion (6AN) are reported. Blood gas analysis (Blood Gas Analyzer; model 170, Corning Medical) was performed on the perfusate and coronary effluent from each heart before and after drug treatment.

### Measurement of Ca^2+^ Currents in Cardiac Myocytes

Single myocytes were isolated using standard protocols described previously [16]. Freshly isolated cells were dispersed in a small chamber mounted on the stage of an inverted microscope (Nikon, Tokyo, Japan) and superfused with Tyrode solution at room temperature (22–25°C). Membrane currents were recorded using the patch clamp technique in the whole-cell configuration with Axopatch 1-A amplifiers and pClamp 9 software (Molecular Devices, CA, USA). Whole cell I_Ca-L_ were recorded using previously published solutions and recording conditions [7], [17]. The pipette solution contained (mM) 135 CsCl, 1.0 MgCl_2_, 5.0 Mg-ATP, 5.0 BAPTA, and 5 HEPES 5 (pH was adjusted to 7.30 with CsOH). After establishing the whole-cell configuration, membrane capacitance (Cm) was estimated by analyzing the transient charges elicited by a 10-mV pulse from a holding potential of −50 mV. Whole cell currents were filtered at 2 kHz, digitized at 10 kHz, and stored on a computer for off-line analysis. To determine I–V relationships, pairs of command pulses were applied at 0.2 Hz. After an initial 20-ms ramp pulse from a holding potential of −80 mV up to −40 mV to inactivate the Na^+^ current, square pulses were applied from the holding potential to −40 mV and increased to +50 mV in 10-mV increments. To obtain steady-state inactivation curves for L-type Ca^2+^ channels, Na^+^ was replaced by isomolar tetraethylammonium (TEA), after which I_Ca-L_ were evoked by 500-ms command pulses beginning from a holding potential of −80 mV and then depolarizing to +40 mV in 10-mV increments. The voltage-dependence of the steady-state inactivation was determined from the peak I_Ca-L_ amplitudes during depolarization to a test potential of 10 mV following 2-s prepulses. Normalized peak I_Ca-L_ amplitudes were plotted vs. V and were fitted by a Boltzmann equation: I/Imax = 1/[+exp (V_1/2_-V)/k], where V_1/2_ is mid-voltage of inactivation and k is the slope of the linear portion of the inactivation curve.

After rupturing the cell membrane and establishing a gigaseal, we waited approximately 10 min for currents to stabilize, after which I_Ca-L_ were recorded for up to 10 min after application of drugs. The rundown of I_Ca-L_ was negligibly small before and during the application of drugs; experiments with large rundown were omitted from our analysis.

### Echocardiography

Cardiac function was assessed in G6PD^deficient^ mice using a Vevo 770 High-Resolution Imaging Systems (Visual Sonics, Ontario, Canada) with a 30 MHz linear array transducer (model 716). Mice were anesthetized with 2.5% isoflurane in oxygen, shaved, and placed on a warming pad. M-mode frames were recorded from the parasternal short axis. Stroke volume (SV) was calculated as the difference between LV end-diastolic volume (EDV) and the LV end-systolic volume (ESV). Ejection fraction (EF) was calculated as EF = [(EDV − ESV)/EDV]×100. Cardiac output (CO) was calculated as CO = SV×HR. EDV and ESV were calculated as: 1.047×EDD^3^ and 1.047×ESD^3^ respectively.

### Glucose-6-Phosphate Dehydrogenase Activity

G6PD activity was measured in myocardial tissue homogenates by following the reduction of NADP^+^ to NADPH as described earlier [11]. NADPH fluorescence was detected as the 460 nm emission elicited by excitation at 340 nm using a Synergy 2 microplate fluorescence detector, BioTek Instruments, Vermont, USA.

### NADPH Levels

To measure NADPH levels, frozen tissue samples were homogenized in extraction medium containing NaOH (0.02 N) and cysteine (0.5 mM). The extracts were then heated at 60°C for 10 min and neutralized with 2 ml of 0.25 M glycylglycine buffer (pH 7.6). The neutralized extracts were centrifuged at 10,000× g for 10 min, after which the supernatants were passed through 0.45 µm Millipore filters. The filtered solutions were used to measure NADPH levels, which were estimated by HPLC method [18] or by using a colorimetric assay kit (Bio Vision, CA, USA).

### Cholesterol Levels

Cholesterol content was measured spectrophotometrically using a cholesterol quantitation kit (Cholesterol/Cholesteryl Ester Quantitation Kit, Bio Vision). Briefly, frozen tissue samples were homogenized in chloroform-Triton X-100, a cholesterol probe was added, and the cholesterol content was estimated in a colorimetric assay, according to the manufacturer's protocol. The protein content of each sample was estimated using a Bradford protein assay, and the cholesterol concentration was expressed as micrograms per milligram of protein.

### Acetyl CoA Levels

Heart and liver acetyl CoA content was estimated by a kit from BioVision.

### Superoxide Levels

To measure superoxide anion (O2−) levels, frozen tissue samples were homogenized in extraction medium containing MOPS (20 mM) and sucrose (250 mM) at 0°C. The extracts were centrifuged at 10,000× g for 10 min, after which the supernatants were then incubated at 37°C for 10 min and O2− was detected based on lucigenin (5 µM) chemiluminescence using a BioTek Syngergy 2 plate luminometer.

### Aconitase Activity

Heart and liver aconitase activity was determined by a kit assay from (OxisResearch, CA, USA).

### Reduced (GSH) and oxidized (GSSG) glutathione Levels

GSH levels were measured using a GSH reductase-based recycling method using a kit from Cayman Chemical Co., MI, USA as previously described [19].

### Glutamic pyruvic transaminase (GPT), γGlutamyltransferase (γGT) and alkaline phosphatase activity

GPT, γGT and alkaline phosphatase activity in wild-type and G6PD^deficient^ liver tissue was determined as previously described [20].

### Serum Metabolic Parameters

Glucose, triglycerides, and free fatty acids were each measured in wild-type and G6PD^deficient^ mouse serum using a colorimetric kit (Wako Diagnostics, Richmond, VA).

### Statistical analysis

All results are expressed as mean ± S.E. Comparisons between groups were made using one-tail unpaired Student's t-tests. Values of P<0.05 were considered significant.

## Results

### Effect of 6AN on myocardial G6PD activity

**Figure 1:**
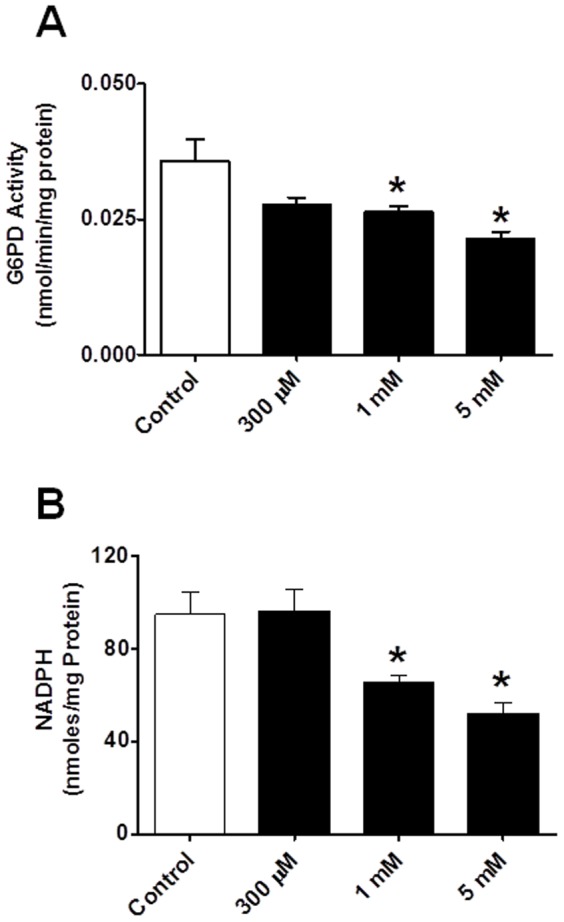
G6PD activity and NADPH levels. Application of 6AN (0.3–5 mM; A and B) for 10 minutes at 37°C to rat hearts significantly reduces both G6PD activity and NADPH levels. *P<0.05 vs. control. Note: G6PD activity was determined from the rate of conversion of NADP^+^ to NADPH in the presence of either glucose-6-phosphate (G6P: 200 µM) substrate and NADPH was measured by HPLC method.

G6PD is the first and rate-limiting enzyme in the oxidative branch of the PPP, which commits glucose to the pathway and is a major source of NADPH in most cells, including cardiac myocytes [7], [12]. We estimated G6PD activity in myocardial homogenates obtained from isolated rat hearts that were left untreated (control) or treated with 6AN (5 mM; n = 5), a competitive blocker that irreversibly inhibits G6PD and decreases O2− in the heart [21], [22]. 6AN dose-dependently reduced G6PD activity (Fig. 1A) and NADPH levels (Fig. 1B).

### Effects of 6AN on cardiac metabolism and contractility, and I_Ca-L_ in cardiac myocytes

**Figure 2:**
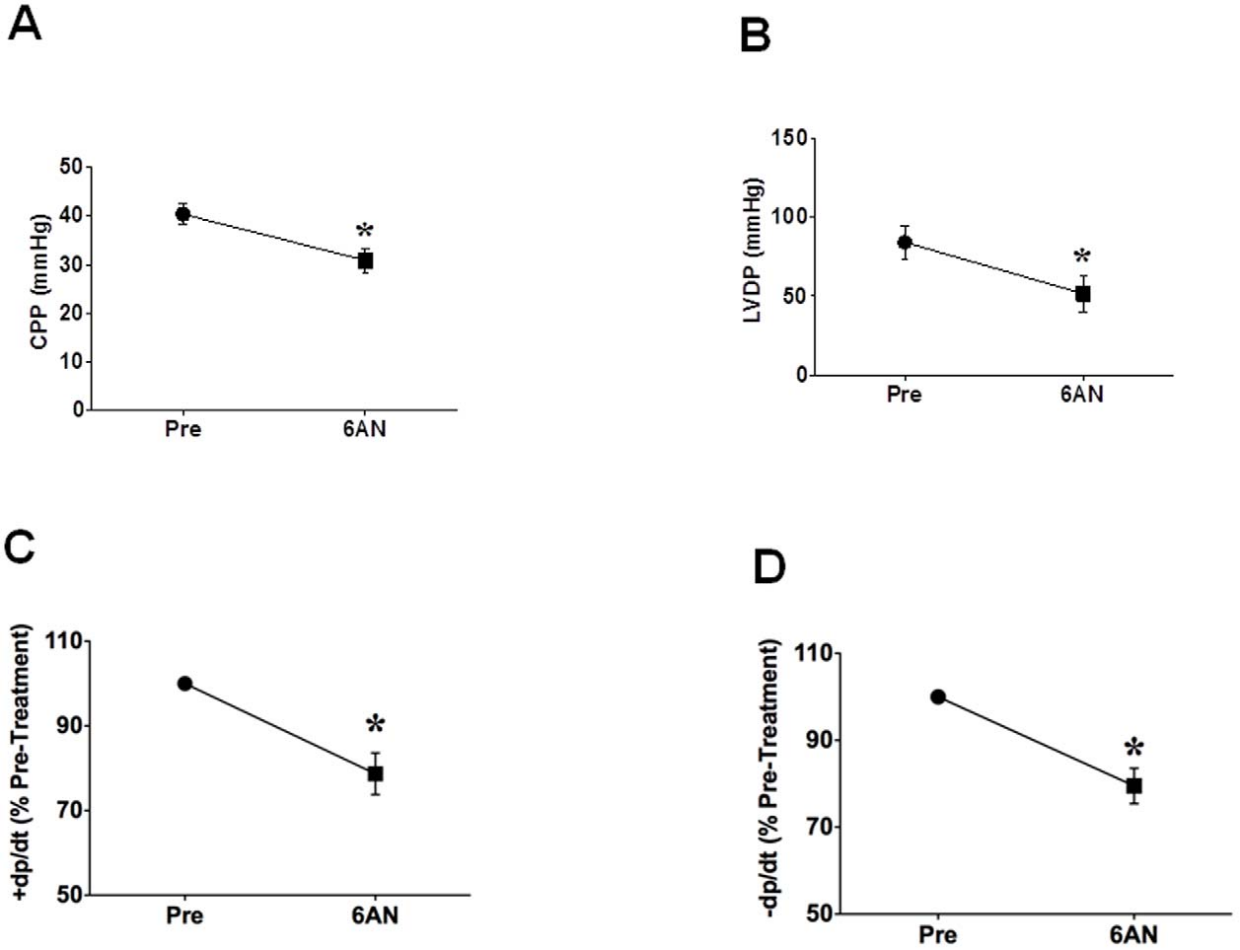
Inhibitory effects of 6AN on myocardial function in isolated rat hearts. 6AN (5 mM) was applied to the hearts in vitro for 10 minutes. A: perfusion of the heart with 6AN decreased coronary perfusion pressure (CPP). B: summary data indicate inhibition of left ventricle developed pressure by 6AN. C and D: summary data indicate inhibition of +dp/dt and −dp/dt by 6AN. Hemodynamic measurements were continuously done during the experiment, but measurement taken just prior to perfusing the heart with 6AN (Pre) and 10 minutes after 6AN perfusion (6AN) are reported. *P<0.05 vs. control (Pre).

**Figure 3:**
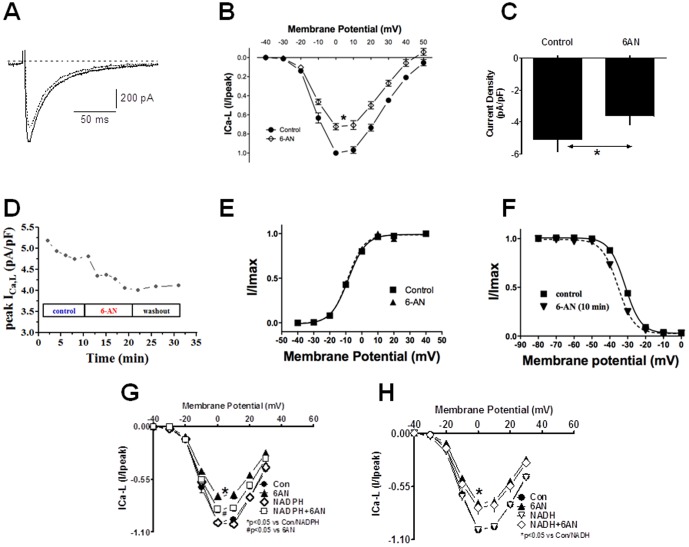
Inhibitory effects of 6AN on I_Ca-L_ in isolated rat cardiomyocytes and effect of NADPH on 6AN-induced inhibition of I_Ca-L_. A and B: I_Ca-L_ were evoked by 500-ms depolarizing pulses to 10 mV applied every 30 s from a holding potential of −80 mV. Cells were superfused with 6AN (5 mM) for 10 minutes after the currents had stabilized. Both the raw traces (A) and I–V relationships (B) show that I_Ca-L_ amplitudes were significantly reduced by 6AN. Currents were normalized to the peak amplitude at 0 mV in the absence of 6AN. Summary data for the normalized current densities (C) and time-course (D) indicate inhibition of I_Ca-L_ by 6AN. Stead-state activation (E) and inactivation (F) curves suggest 6AN had no significant effect on activation and inactivation kinetics. Summary data for the normalized current densities in cells dialyzed with NADPH (100 µM) and exposed to 6AN are shown. The I–V relationships indicate that I_Ca-L_ amplitudes were significantly reduced by 6AN (G), but that dialysis of NADPH partially reversed their inhibitory effect. In contrast NADH had no effect on 6AN-induced inhibition of I_Ca-L_ (H). Currents were normalized to the peak amplitude at 0 mV in the absence of 6AN. *P<0.05 vs. control. ^#^P<0.05 vs 6AN.

To determine if perfusion of the heart with 6-AN alter metabolism, we performed blood gas analysis. The inhibition of G6PD by perfusion of the hearts in constant flow mode with 6-AN (5 mM; n = 5) significantly (P<0.05) increased pCO_2_ and decreased pH (Table 1). Concomitantly, 6-AN decreased coronary perfusion pressure (CPP), LVDP and ±dp/dt (Fig. 2). 6AN was washed out from the hearts by perfusing normal Krebs solution. CPP, LVDP and ±dp/dt did not decrease further but remained stable during 60 minutes of wash out period. We next recorded L-type Ca^2+^ currents in the absence and presence of the G6PD inhibitor 6AN (5 mM; n = 8). We found that application of 6AN to the extracellular side of isolated cardiac myocytes significantly reduced I_Ca-L_ amplitude (Fig. 3A, 3B) and current density at 0 mV (Fig. 3C), and washing out 6AN with normal Tyrode solution stopped 6AN-evoked decrease and in some cases partially reversed 6AN-evoked reduction of I_Ca-L_ (Fig. 3D). 6-AN did not affect the I–V relationship (Fig. 3B) and steady-state activation curve (Fig. 3E), but slightly shifted the steady-state inactivation from −31.480±1.10 to −35.43±0.96 (NS; Fig. 3F).

**Table 1:** Blood gas analysis of coronary perfusate.

	Buffer	pre-6AN	post-6AN	P-Value (n = 5)
pH	7.43±0.02	7.39±0.01	7.33±0.02	P = 0.0082 vs pre-6AN
pCO_2_ (mmHg)	36.7±1.2	40.8±1.0	45.8±1.3	P = 0.017 vs pre-6AN
pO_2_ (mmHg)	629±12	227±17	249±21	NS
Na^+^ (mmol/L	140.7±2.3	142.0±0.4	143.0±0.5	NS
K^+^ (mmol/L)	5.6±0.1	5.6±0.02	5.8±0.09	NS
Ca^2+^ (mmol/L)	2.07±0.04	2.14±0.03	2.14±0.01	NS
Glucose (mg/dl)	200±5	197±2	196±1	NS
Lactate (mmol/L)	0±0	0±0	0±0	NS

### Effects of increasing intracellular NADPH on 6AN-induced suppression of I_Ca-L_ in cardiac myocytes

Finally, to assess the extent to which a reduction in the NADPH redox potential caused by inactivation of G6PD led to inhibition of L-type Ca^2+^ channel activity, we tested the effects of exogenously increasing intracellular NAD(P)H on 6AN-induced inhibition of L-type Ca^2+^ channel activity in single ventricular myocytes. To raise intracellular levels, either NADPH (100 µM; n = 5) or NADH (100 µM; n = 5) was dialyzed into the cells from the pipette solution. Notably, the exogenous NADPH inhibited the reductions in I_Ca-L_ amplitude and I/I_peak_ evoked 6AN (Fig. 3G). By contrast, the inhibitory effects of 6AN on L-type Ca^2+^ channel activity were unaffected by NADH (Fig. 3H).

### Effects of G6PD deficiency on cardiac reactive oxygen species generation and metabolism

**Figure 4:**
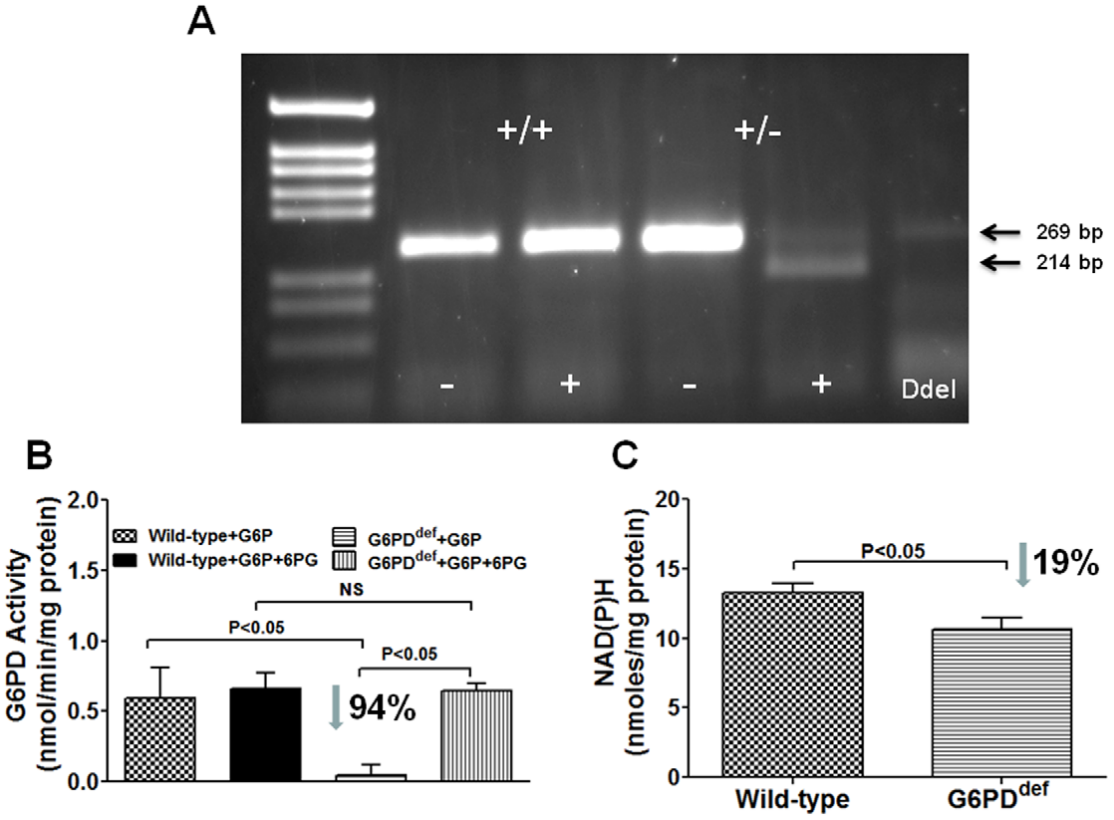
G6PD activity and NADPH levels in G6PD^deficient^ mice heart. A: DNA prepared from tails snips using Viagen DirectPCR reagent. DNA amplification done using Tfi DNA polymerase (Invitrogen). Reaction products were incubated at 37C for 1 hour in the absence or presence of Dde1 before being loaded onto a 1.5% Agarose gel. Presence of the Dde1 site is indicative of the mutation in the 5′-UTR G6PD sequence and shifts the 269 bp reaction product down to 214 bp. Size markers are Pgem from Promeage. B; G6PD activity was determined from the rate of conversion of NADP^+^ to NADPH in the presence of either glucose-6-phosphate (G6P) or G6P+6-phosphogluconate (6PG) substrates. C; NADPH levels in wild-type and G6PD^deficient^ mice hearts measured by a colorimetric method are compared.

**Figure 5:**
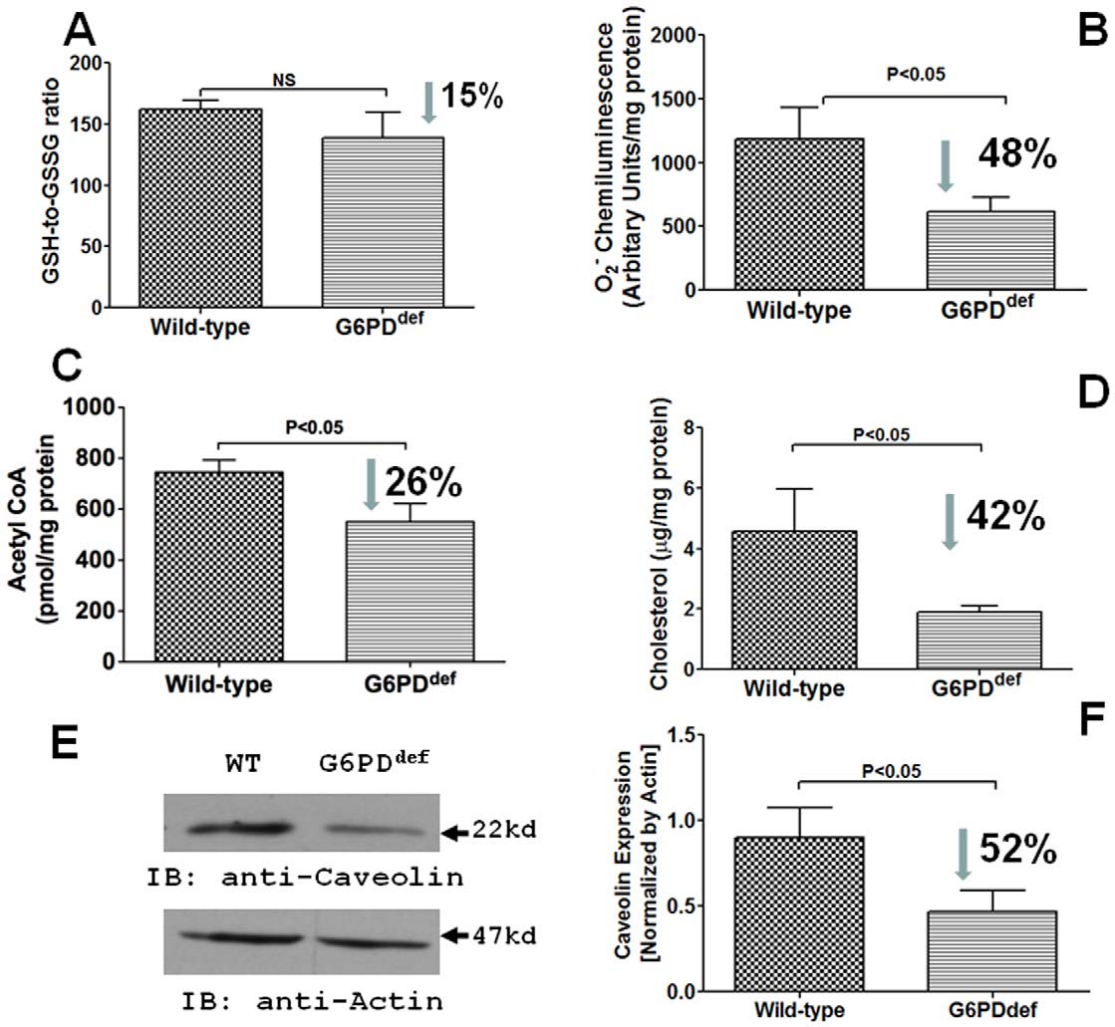
Redox, acetyl CoA and cholesterol levels in G6PD^deficient^ mice hearts. Metabolites produced from NADPH-dependent reaction were estimated in wild-type and G6PD^deficient^ mice hearts. A and B: G6PD deficiency decreased reduced glutathione-to-oxidized glutathione (GSH-to-GSSG; A) ratios determined by colorimetric assay and superoxide (O2−; B) estimated by lucigenin (5 µM) chemiluminescence method. Concurrently, G6PD deficiency lowered NADPH-dependent free fatty acid β-oxidation, which produces acetyl CoA (C), cholesterol synthesis (D), and caveolin (E–F) in the hearts as well.

G6PD-derived NADPH regulates superoxide anion (O2−) production, reduced glutathione levels, cholesterol and free fatty acid metabolism in the cell. To determine if G6PD deficiency evokes maladaptive response in the heart, we estimated O2− and metabolic products in G6PD^deficient^ (n = 9) and wild-type (n = 8) mice heart. Presence of the Dde1 site is indicative of the mutation in the 5′-UTR G6PD sequence and shifts the 269 bp PCR product down to 214 bp (Fig. 4A), and in these mice hearts G6PD activity was reduced by 80–90% (Fig. 4B. However, surprisingly NADPH was only decreased (P<0.05) by 19% of the wild-type (Fig. 4C). Consistently, NADPH-dependent oxidative stress markers GSH-to-GSSG ratio and O2− were also decreased by 48% of the wild-type (Fig. 5A–B), while aconitase activity (Wild-type: 374.90±28.28 and G6PD^def^: 367.90±23.02 mU/mg Protein), which is inhibited by over production of O2−, was unchanged in G6PD^deficient^ hearts as compared to the wild-type hearts. Moreover, acetyl CoA and cholesterol that requires NADPH for their synthesis and metabolisms were concurrently decreased in G6PD^deficient^ hearts (Fig. 5C–D). We also found that less (52%) caveolin expression in G6PD^deficient^ as compared to wild-type hearts (Fig. 5E–F).

### Effects of G6PD deficiency on liver reactive oxygen species generation and metabolism

Glucose metabolism mainly occurs in the liver, and G6PD activity is higher in the liver than the heart. Also, G6PD-derived NADPH is utilized in the liver for cholesterol synthesis and fatty acid metabolism. Therefore, to confirm our results from G6PD^deficient^ hearts, we performed biochemical analysis in the liver tissue as well. Likewise G6PD^deficient^ livers had lower superoxide and cholesterol (Table 2). Additionally, hepatic GPT and γGT activity were higher (P<0.05) in G6PD^deficient^ than wild-type mice. G6PD deficiency did not affect blood glucose, free fatty acid and triglyceride levels (Table 3) or glucose up-take between over-night fasting wild-type and G6PD^deficient^ mice determined by intraperitoneal glucose tolerance test was not different (data not shown).

**Table 2:** NADPH, NADPH-dependent metabolites, and enzyme activity in Glc-6-PD^deficient^ liver.

Parameters	Wild-type (n = 5)	G6PD^def^ (n = 5)	P-value
NADPH (nmol/mg Protein)	3239±1743	1859±1215	NS
Superoxide (AU/mg Protein)	490.5±128.0	116.6±42.2	P = 0.0098
Cholesterol (µg/mg Protein)	4.40±1.13	1.33±0.11	P = 0.0239
Acetyl CoA (nmol/mg Protein)	9.16±1.90	20.47±7.10	P = 0.0774
Aconitase activity (mU/mg Protein)	132.50±4.32	129.10±5.81	NS
Alkaline Phosphatase activity (IU/L)	86.18±4.68	90.81±3.99	NS
Glutamic pyruvic transaminase activity (IU/L)	450.6±13.9	491.8±11.7	P = 0.0183
γGlutamyltransferase activity (IU/L)	1.74±5.77	10.42±1.88	P = 0.0682

**Table 3:** Serum free fatty acid, triglycerides and glucose levels in G6PD^deficient^ mice (n = 5).

Strain	FFA (mM)	TG (mg/dL)	Glucose (mg/dL)
Wild-type	0.55±0.08	347.80±43.71	223.86±8.58
G6PD^def^	0.48±0.03	312.88±28.77	237.99±10.04
P-value	NS	NS	NS

### Effects of G6PD deficiency on cardiac function

**Figure 6:**
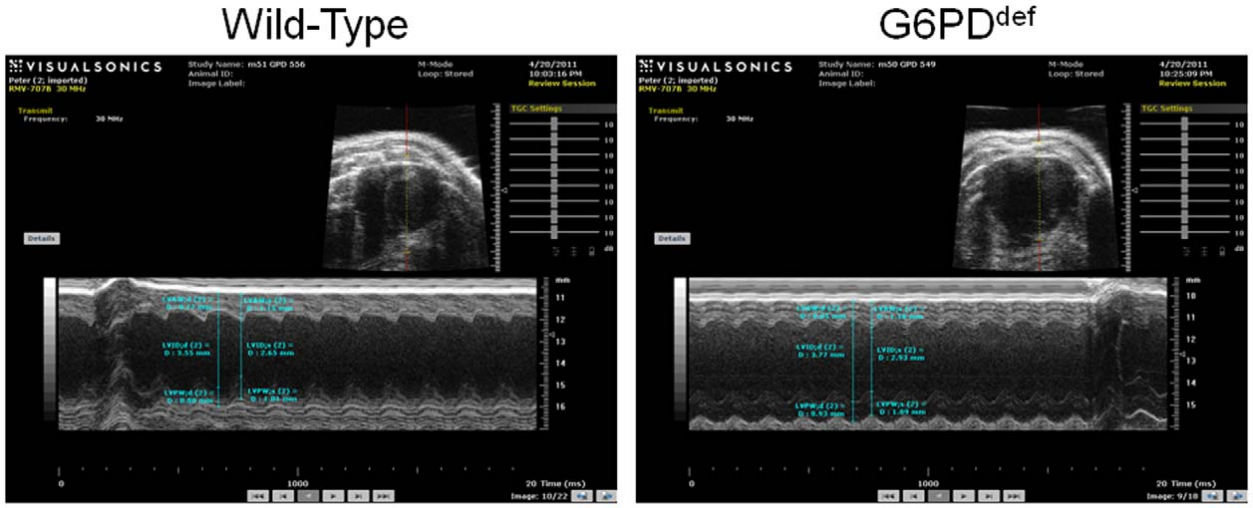
M-mode echocardiography performed on wild-type and G6PD^deficient^ mice hearts is shown.

Next, we used echocardiography to evaluate LV structure and function in 17- to 18-wk-old wild-type and G6PD^deficient^ mice (Fig. 6). The data is summarized in Table 4, LV diastolic volume, end-diastolic diameter, stroke volume, cardiac output and cardiac index increased (P<0.05) in G6PD^deficient^ mice. On the other hand, there was no significant LV failure (unchanged LV ejection faction and fraction shortening) and there were no premature mortalities in G6PD^deficient^ group.

**Table 4:** Summary data of M-mode echocardiography.

Parameter	Wild-Type (n = 8)	G6PD^def^ (n = 10)	P-value
Age	17–18 wks	17–18 wks	
Body Weight (g)	31.41±0.81	29.45±0.77	NS
Hear Rate (beats/min)	448±13	450±07	NS
LV Diastolic Volume (µL)	58.46±5.56	69.54±3.01	P = 0.0412
LV Systolic Volume (µL)	31.28±4.54	37.21±2.62	NS
End Diastolic Diameter (mm)	3.80±0.12	4.04±0.06	P = 0.0322
End Systolic Diameter (mm)	3.05±0.14	3.27±0.08	NS
Stroke Volume (SV: µL)	27.18±1.22	32.33±1.67	P = 0.0149
Cardiac Output (SV*HR)	12179±628	14563±765	P = 0.0167
Cardiac Index (SV*HR*BSA^−1^)	389535±22735	495161±24673	P = 0.0036
Absolute Wall Thickness (mm)	1.75±0.11	1.58±0.08	NS
Relative Wall Thickness	0.47±0.04	0.39±0.02	NS
Fraction Shortening	0.20±0.01	0.19±0.01	NS

HR = Heart Rate; BSA = Body Surface Area.

**Figure 7:**
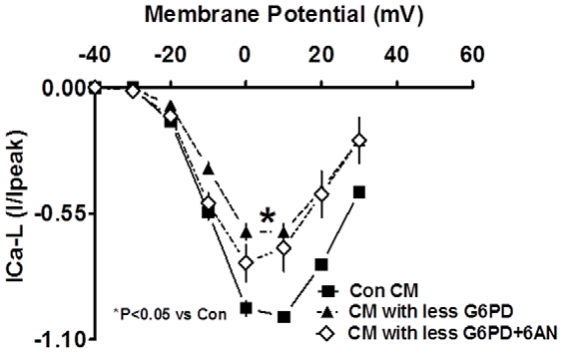
I–V relationships show that I_Ca-L_ amplitudes were significantly reduced in the cardiac myocytes (CM) with less G6PD than the control CM.

I_CaL_ were reduced (P<0.05; n = 3–4) in cardiac myocytes that had 60–80% less G6PD than normal, and currents were not reduced further by application of 6AN (5 mM) to those cells (Fig. 7).

## Discussion

It is well known that steroids can affect ion channel activity and alter the ionic homeostasis within blood vessels and the myocardium. Hormones inhibit evoked elevations in intracellular Ca^2+^ and activate K^+^ efflux in both vascular smooth muscle and cardiac myocytes. In particular, 17β-estradiol and testosterone inhibit the activity of stably expressed voltage-gated Ca^2+^ channels (L- and T-type Ca^2+^ channels) in isolated guinea-pig atrial [1] and ventricular myocytes [2], A7r5 cells [6], and HEK293 cells [5]. And it has been proposed in recent studies that by regulating I_Ks_ and I_Ca-L_, testosterone modulates cardiac repolarization, thereby potentially contributing to the control of QTc intervals [3]. Others and we have demonstrated that epiandrosterone and DHEA also reduce myocardial contractility and the contractility of isolated cardiac myocytes by inhibiting I_Ca-L_ and diminishing Ca^2+^ transients [7], [12]. However, the mechanisms by which these steroids inhibit L-type Ca^2+^ channel activity remained unclear.

Evidence from some studies suggests that, like some Ca^2+^ channel blockers, steroids directly bind the channel protein and inhibit L-type Ca^2+^ currents by accelerating channel inactivation and stabilizing the channels in the inactivated state [23]–[25]. Consistent with that idea, certain steroid metabolites are able to influence neuronal excitability directly by acting at the membrane [26]. Alternatively, others have suggested that testosterone suppresses I_Ca-L_ and facilitates cardiac repolarization by activating the c-Src-Akt-NOS3 pathway and NO synthesis [3]. By contrast, epiandrosterone and DHEA both suppress I_Ca-L_ and Ca^2+^ transients in isolated cardiac myocytes presumably through an NO-independent mechanism, since epiandrosterone decreases NO_x_ production and oxidation of glutathione by DHEA decreases Ca^2+^ transients [7], [12]. Instead, evidence suggests DHEA binds to the ternary G6PD-coenzyme-substrate complex(es), thereby inhibiting the enzymes activity [8]. This led us to postulate, that epiandrosterone and DHEA suppresses L-type Ca^2+^ channel activity and reduces Ca^2+^ influx, presumably by inhibiting G6PD activity and reducing NADPH production. If true, then 6AN, a nonsteroidal G6PD inhibitor that inactivates the enzyme by competing with endogenous NADP^+^ for its binding site [15], should suppress L-type Ca^2+^ channel activity and cardiac contractility.

Interestingly, we found that 6AN, which inhibits the PPP in hearts [21], [22], [27], reduced G6PD activity and NADPH production and increased pCO_2_. This finding suggests that, at least, a small portion of glucose was oxidized in the PPP in normal hearts and inhibition of G6PD by 6-AN increased glucose oxidation in the glycolytic pathway. Concurrently, perfusion of the heart with 6-AN decreased coronary perfusion pressure, and suppressed cardiac myocyte I_Ca-L_, and myocardial contractility (±dp/dt). A similar inhibition (35.5±3.2% as compared to untreated controls) of I_Ca-L_, was observed in the presence of BayK 8644 (1 µM), a L-type Ca2+ agonist. Although additional mechanisms/signaling pathways like, increased pCO_2_ or decreased pH that decrease L-type Ca^2+^ channel function, cannot be ruled out these results suggest that the inhibition of cardiac myocyte I_Ca-L_ and contractility induced by 6AN is mediated at least in part by oxidization of NADPH.

We found that dialyzing NADPH, but not NADH, into cells from the pipette solution partially reversed 6AN-induced suppression of I_Ca-L_. Furthermore perfusion of dithiothreitol (2 mM) partially decreased suppression of I_Ca-L_ evoked by G6PD inhibitor. We therefore propose that G6PD-derived NADPH production may play a novel role in regulating the activity of L-type Ca^2+^ channels. Although mechanism by which NADPH redox modulates I_Ca-L_ is unclear, we suggest that it may involve changes in glutathione redox, which is directly modulated by NADPH. Indeed, glutathione redox has been shown to regulate cardiac myocyte L-type Ca^2+^ channels through oxidation of the cysteine sulfhydryl group present on the channel's α_1c_ subunit [28], [29]. In addition, H_2_O_2_ is known to regulate sensitivity of Ca^2+^ channel activity to β-adrenergic agonist stimulation and scavenging of H_2_O_2_ by catalase significantly enhances the sensitivity of I_Ca-L_ to isoproterenol [30]; consequently, a reduction in NADPH, which controls NADPH oxidase-derived H_2_O_2_ production in both cardiac [31] and smooth muscle [32], also could contribute to L-type Ca^2+^ channel inhibition.

Inhibition of G6PD by DHEA has been shown to deplete cytosolic glutathione levels, thereby causing contractile dysfunction through dysregulation of Ca^2+^ homeostasis [12], and inhibition of G6PD by epiandrosterone has been shown to evoke suppression of I_Ca-L_ by decreasing the amplitude and shifting steady-state inactivation curve to hyperpolarizing potentials [7]. Unlike, epiandrosterone and dihydropyridine class of L-type Ca^2+^ channel blockers, 6AN suppressed the channel activity by decreasing current amplitude without significantly affecting steady-state activation or inactivation state. Therefore, these results suggest that steroids exert their inhibitory effects through other mechanisms in addition to G6PD-dependent redox changes.

Next, to determine whether G6PD deficiency affects cardiac metabolism and heart structure and function; we performed biochemical tests and M-mode echocardiography on 17–18 wk old G6PD^deficient^ and age/sex matched wild-type mice. Contrary to our expectation, NADPH levels in G6PD^deficient^ mice heart (P<0.05) were slightly reduced as compared to a significant loss of G6PD activity. These findings were a bit surprising. Nonetheless, we speculate that due to loss of a major NADPH synthesis pathway cells might have up-regulated compensatory source of NADPH such as, malic enzyme, to protect the heart tissue from oxidative damage in G6PD^deficient^ mice. Consistently, hearts from G6PD^deficient^ mice had a small decrease (NS) in GSH-to-GSSG ratios but a significant reduction in O2− levels. G6PD-derived NADPH is a substrate for O2− producing NADPH oxidase in the heart [19]–[22], and therefore a decrease in O2− in G6PD^deficient^ mice hearts may not be unprecedented. Additionally, we also found that G6PD^deficient^ mice hearts had lower cholesterol and acetyl CoA content. Cholesterol synthesis via HMG-CoA and fatty acid synthesis and catabolism, which produces acetyl CoA from polyunsaturated fatty acid in liver and heart mitochondria is catalyzed by NADPH [33], [34]. Therefore, our findings reflect that G6PD insufficiency in heart and liver from G6PD^deficient^ mice may be a primary cause for decrease in cholesterol anabolism and fatty acid β-oxidation. Both cholesterol and fatty acid are involved in several cell transactions like, plasma membrane formation, energy metabolism, and signaling. Cholesterol is required to maintain membrane stability and signaling, and reduction in membrane cholesterol reduces basal and α-agonists evoked I_Ca-L_ in cardiac myocytes [35]. Concurrently, expression of caveolin, a protein marker for cholesterol enriched caveolae specialized lipid raft/micro domain in the myocytes, was significantly reduced. As cholesterol and caveolin is required to maintain the integrity of caveolae structure, it is reasonable to speculate that reduced cholesterol/caveolin might have disrupted caveolae and impaired signal transduction in G6PD^deficient^ heart. Whereas, fatty acid oxidation is a primary source of energy in the heart tissue, and reduction in fatty acid β-oxidation leads to abnormal myocardial function [36]. Consistent with this notion, we have previously found that membrane depolarization and G-protein coupled receptor mediated vascular smooth muscle contractions are significantly reduced in G6PD^deficient^ animals [37]. We, therefore, propose that G6PD-derived NADPH is required to regulate lipid metabolism and redox-dependent signaling in the normal heart and insufficiency of G6PD may affect heart function.

In echocardiography, we found that end-systolic diameter, fraction shortening and ejection faction in G6PD^deficient^ mice were unchanged as compared to the wild-type mice. Intriguingly, however, the LV end-diastolic volume and diameter and stroke volume was increased in G6PD^deficient^ mice. These findings collectively suggest that the heart function was slightly altered in G6PD^deficient^ mice presumably due to long term remodeling and are somewhat similar to those of Jain et al [12], who reported a slight but insignificant increase in end-diastolic diameter and significant increase in end-systolic diameter with not much change in fraction shortening in 24 wks old mice. But they found a significant decrease in fraction shortening in older (36 wks) mice. These findings imply that G6PD deficiency evokes remodeling of the left heart in young animals, and as they grow older it compromises cardiac function perhaps through increasing susceptibility to oxidative injury or by impairing intracellular calcium transport in cardiomyocytes. Although these changes in heart function are not too severe in normal conditions, whether cardiac dysfunction is exacerbated in pathologies of the heart in G6PD^deficient^ mice remains to be seen. Several epidemiological studies suggest that the individuals harboring a Mediterranean-type mutation are less likely to have cardiovascular diseases, including ischemic heart disease and heart failure [13], [38]. Supporting this notion up-regulation of G6PD expression and activity has been associated with heart failure [19], [39]–[42], while it has been proposed for a long time that G6PD-derived ribose sugar promotes the development of hypertrophy/compensated heart failure [43], [44]. These studies, therefore, suggest G6PD is a double-edged sword as too little or too much G6PD can profoundly affect intracellular redox potential and ROS content, which can be both beneficial and/or detrimental to the heart function.

In conclusion, the present findings provide evidence that modulation of glucose metabolism via the PPP alters cellular redox potentials, L-type Ca^2+^ channel activity, and myocardial function, which may have implications for the development of cardiovascular diseases or alternatively could be one mechanism by which steroid hormones such as DHEA protect the heart. Nonetheless, more detailed studies are required to determine the right balance of G6PD and G6PD-dependent metabolites for maintaining a healthy heart.
